# Global public health must end the neglect of oral health

**DOI:** 10.1093/eurpub/ckac129.371

**Published:** 2022-10-25

**Authors:** H Benzian, S Loistl

**Affiliations:** 1 College of Dentistry, New York University, New York, USA; 2 Radboud Institute for Health Science, Radboud University, Nijmegen, Netherlands

## Abstract

Oral diseases are among the most common health problems and constitute major public health challenges across countries and health systems worldwide. The main diseases dental caries, severe periodontal disease, oral cancer and toothlessness affect populations everywhere and show significant social gradients - poor, disadvantaged, rural, or otherwise vulnerable groups suffer from a much higher burden, while at the same time having more difficulties to access prevention and care. Similarly, low- and middle-income countries have the highest prevalence of oral diseases while their healthcare systems are ill-equipped to address the challenges. Due to the predominant clinical care model of dentistry that favours an interventionist approach requiring costly technology provided by a dentist, many health systems and large population segments are not able to afford even basic oral health care. Moreover, oral health has been largely absent from the global health arena for the last 20 years leading to faltering political priority and attention. This has changed recently with the convergence of several initiatives of global relevance, supported by advocacy of the wider oral health community. Following the first ever paper series on oral health in The Lancet, a Lancet Commission on Oral Health was established in 2019, which sparked a group of WHO member states to embark on a remarkable policy development process dedicated to oral health. We now have a Global Strategy for Oral Health and will soon have a Global Action Plan complemented by a comprehensive Monitoring Framework. In addition, work on defining Best Buys in Oral Health and other supportive workstreams are under way. The presentation will provide an introduction and overview to these global policy guidance frameworks that are shaping a new reform agenda for oral health as part of NCDs, Universal Health Coverage and the SDGs towards 2030. Particular implications for public oral health in Europe will be discussed.

